# A Comprehensive Review of Pesticide Residues in Peppers

**DOI:** 10.3390/foods12050970

**Published:** 2023-02-24

**Authors:** Jae-Han Shim, Jong-Bang Eun, Ahmed A. Zaky, Ahmed S. Hussein, Ahmet Hacimüftüoğlu, A. M. Abd El-Aty

**Affiliations:** 1 Natural Products Chemistry Laboratory, Biotechnology Research Institute, Chonnam National University, 77 Yongbong-ro, Buk-gu, Gwangju 500-757, Republic of Korea; 2 Department of Food Science and Technology, Chonnam National University, Gwangju 61186, Republic of Korea; 3 Department of Food Technology, Food Industries and Nutrition Research Institute, National Research Centre, Cairo 12622, Egypt; 4 Department of Medical Pharmacology, Medical Faculty, Ataturk University, Erzurum 25240, Turkey; 5 Vaccine Development Application and Research Center, Ataturk University, Erzurum 25240, Turkey; 6 Department of Pharmacology, Faculty of Veterinary Medicine, Cairo University, Giza 12211, Egypt

**Keywords:** pepper, pesticide residue, monitoring, analytical approach, maximum residue limit, dissipation pattern

## Abstract

Pesticides are chemicals that are used to control pests such as insects, fungi, and weeds. Pesticide residues can remain on crops after application. Peppers are popular and versatile foods that are valued for their flavor, nutrition, and medicinal properties. The consumption of raw or fresh peppers (bell and chili) can have important health benefits due to their high levels of vitamins, minerals, and antioxidants. Therefore, it is crucial to consider factors such as pesticide use and preparation methods to fully realize these benefits. Ensuring that the levels of pesticide residues in peppers are not harmful to human health requires rigorous and continuous monitoring. Several analytical methods, such as gas chromatography (GC), liquid chromatography (LC), mass spectrometry (MS), infrared spectroscopy (IR), ultraviolet–visible spectroscopy (UV–Vis), and nuclear magnetic resonance spectroscopy (NMR), can detect and quantify pesticide residues in peppers. The choice of analytical method depends on the specific pesticide, that is being tested for and the type of sample being analyzed. The sample preparation method usually involves several processes. This includes extraction, which is used to separate the pesticides from the pepper matrix, and cleanup, which removes any interfering substances that could affect the accuracy of the analysis. Regulatory agencies or food safety organizations typically monitor pesticide residues in peppers by stipulating maximum residue limits (MRLs). Herein, we discuss various sample preparation, cleanup, and analytical techniques, as well as the dissipation patterns and application of monitoring strategies for analyzing pesticides in peppers to help safeguard against potential human health risks. From the authors’ perspective, several challenges and limitations exist in the analytical approach to monitoring pesticide residues in peppers. These include the complexity of the matrix, the limited sensitivity of some analytical methods, cost and time, a lack of standard methods, and limited sample size. Furthermore, developing new analytical methods, using machine learning and artificial intelligence, promoting sustainable and organic growing practices, improving sample preparation methods, and increasing standardization could assist efficiently in analyzing pesticide residues in peppers.

## 1. Introduction

Peppers are an important part of many diets worldwide due to their nutritional value. Peppers are a rich source of vitamins and minerals, including vitamins C and A and potassium [1]. They are also high in antioxidants and phytochemicals [2], which are associated with many health benefits, including boosting the immune system [3], maintaining healthy skin and eyesight [4], reducing the risk of chronic diseases, such as cancer and cardiovascular disease [3], and managing blood sugar levels [4]. Furthermore, peppers have a distinctive flavor [5] and can be used in various dishes, including salads, sandwiches, and cooked dishes. They can also add color and flavor to various dishes, including soups, stews, and stir-fries. Moreover, peppers are an important part of the cuisine and culture of many countries worldwide. They are often key ingredients in traditional dishes, such as curries, stews, and sauces. In the Republic of Korea, chili peppers are frequently used in traditional cuisines, such as kimchi [6]. They are available in various forms, including fresh, cooked, dried powder, processed, and frozen, and there are various types of pepper, including sweet peppers and hot peppers. Some common examples include bell peppers, banana peppers, Cubanelle peppers, Jalapeño peppers, Habanero peppers, and Serrano peppers [7,8]. Peppers are also an important source of income for many small farmers, particularly in developing countries where they are grown for local consumption and export and contribute to the economic development of communities. According to the Food and Agriculture Organization of the United Nations (FAO), the worldwide production of pepper was approximately 540,000 metric tons in 2020. The top five pepper-producing countries worldwide are listed in the following order: Vietnam, Indonesia, Brazil, India, and Malaysia [9].

Pepper leaves can be used as an ingredient in a type of Korean dish called *Namul Namul* refers to dishes made with seasoned vegetables and herbs that serve as side dishes or as part of a larger meal [10]. In general, they are not typically consumed as food. However, they can be used in traditional medicine in some cultures. Pepper leaves have been used in traditional remedies to treat a range of ailments, including digestive disorders and respiratory diseases. However, pepper leaves have not been extensively studied for medicinal purposes, and their safety and effectiveness have not yet been established.

The quantity and safety of agricultural products are intimately linked to public health, social stability, and sustainable development. Therefore, this topic has gained increasing concern among the general public, health authorities, and the scientific community [11]. The World Health Organization (WHO) states that fruits and vegetables are crucial to a healthy diet. Reduced consumption of fruits and vegetables could correlate with poor health and a higher risk of noncommunicable diseases.

Many pests, including insects, fungi, and weeds, can affect pepper plants. Insects, such as aphids, whiteflies, and mites, can feed on the leaves and stems of pepper plants, causing damage and reducing yields. Insects can also transmit diseases, such as viruses and bacteria, to pepper plants. Fungal diseases in pepper plants, such as anthracnose and blossom end rot, can cause symptoms such as leaf and fruit discoloration, wilting, and plant tissue death. Additionally, weeds can compete with pepper plants for light, water, and nutrients: reducing the growth and yield of pepper plants. Weeds can also harbor pests and diseases that can affect pepper plants.

Insecticides, herbicides, and fungicides are often used to help increase the yield of pepper crops by reducing the damage caused by pests. This could improve farmers’ profitability in terms of pepper production [12]. However, it is important to note that the use of pesticides can also have negative impacts, including risks to human health and the environment [13]. The WHO has reported that approximately three million cases of pesticide poisoning occur annually across the globe, of which 220,000 cases are fatal [14]. As a result of population increase and rapid urbanization, the use of pesticides is also continuously rising [15]. Pesticides can remain on pepper surfaces after their application, and consuming peppers with high pesticide residues can harm human health. In addition, pesticide use can also negatively impact nontarget species, such as birds and bees, and contribute to the decline in these species [16]. It can also lead to the development of resistance in pests, requiring the use of even more pesticides. To mitigate the negative impacts of pesticides, it is vital to use them only when necessary and to follow proper application and safety guidelines. It is also important to monitor and control the levels of pesticide residues in peppers to ensure that they are safe for human consumption.

The intake of raw and cooked vegetables, such as peppers, is one of the most common pesticide exposure routes [17]. The impact of pesticides on the nutritional value of peppers depends on several factors, including the type and amount of pesticide used, the length of time the pesticides remained on the peppers, and their overall nutritional content. In general, pesticides can potentially reduce the nutritional value of peppers by decreasing the levels of certain nutrients, such as vitamins and minerals [18]. Some pesticides may also have toxic effects on beneficial microorganisms in the soil, which could affect the overall nutrient content of peppers.

The current review discusses different extraction and analytical procedures that are used to determine pesticide residues in peppers. Additionally, dissipation patterns and monitoring strategies are also reviewed. A conclusion and potential future perspectives are proposed based on the authors’ viewpoints.

## 2. Maximum Residue Limits

Maximum residue limits (MRLs) are regulatory limits that are set for the levels of pesticide that are allowed to remain on food crops, including peppers, after the application of plant production products and preharvest intervals. MRLs are set to protect human health by ensuring that pesticides on food crops are below the levels that could potentially be harmful. MRLs for pesticides can vary between countries, as each country may have different regulations and guidelines for pesticide use. Some countries may have specific MRLs for particular pesticides in peppers, while others may have more general MRLs for specific pesticides in all food crops. MRLs are typically set based on the results of toxicological assessments, which evaluate the potential health risks of different pesticides [19]. MRLs are typically set at levels well below those that could potentially harm human health. It is important to note that MRLs are not intended to measure pesticide safety.

Disagreements over permissible levels between nations could impede trade globally, thus highlighting the urgent need for MRL standardization. The European Union (EU) and the Codex Alimentarius Commission of the Joint Food and Agricultural Organization of the United Nations (FAO)/WHO [20] have established reference MRLs. Each country uses one of two strategies to limit pesticide residues in agricultural commodities: (a) the regulatory monitoring of agricultural raw materials, measuring the residual levels of particular matrices following the MRL [21,22]; or (b) whole diet research, which analyzes the foods people eat to estimate their dietary intake of pesticides [23,24,25]. A viable approach is required to identify and measure residues at a level equal to or lower than the MRL (Table 1) and verify the identification of substances in agricultural products for research and regulatory purposes. The fundamental steps in multi-residue methods (MRMs) and single-residue methods (SRMs) are essentially the same. To monitor or screen different kinds of pesticides in specific products, MRMs are typically used. By contrast, SRMs are often used for substances that cannot be determined by MRM methods and require specific procedures for sample preparation and determination [26,27,28,29]. Consequently, a suitable analytical technique should be established to quantitatively identify the levels of pesticides in peppers for safety and dietary risk assessment.

## 3. Sample Pretreatment and Extraction Methods

Analytical methods are vital for estimating MRLs, from sample homogeneity to instrument detection limits. In pesticide research, substantial efforts have been undertaken to create and evaluate analytical techniques and procedures. Suppose the experimental sample is too small to accurately represent the initial batch or unit. In this case, applying sophisticated analytical tools and procedures would be expensive, time-consuming, and inefficient and could provide data that are challenging to understand instead of useful findings [48,49]. Consequently, efficient sample preparation is essential for accurately determining pesticide residues in foods with complex matrices [50].

The distribution of pesticide residues in/on crops is diverse. Thus, the sample needs to be completely homogenized. The matrix components are frequently coextracted with specific pesticides after obtaining a suitably homogeneous sample. Notably, more than 2500 natural compounds are found in paprika [51], which may hide the detection of some pesticide residues. In most conventional methods, the samples are extracted with acetonitrile and/or acetone. NaCl was added to the aqueous phase (either as a saturated solution or in solid form) to broaden the polarity range. Afterward, the extract was partitioned with nonpolar solutions (dichloromethane [DCM] or DCM/petroleum ether) to eliminate water and coextracts (e.g., pigments, phenols, and tannins obtained during liquid–liquid partitioning). The utilization of DCM in the liquid–liquid partitioning process was prohibited in 1980 because of the harmful impacts of chlorinated solvents on the environment and human health [52]. Therefore, many attempts have been made to substitute DCM or remove the liquid–liquid partitioning phase. In this context, a cyclohexane/ethyl acetate combination (1:1, *v*/*v*) was employed instead of DCM/petroleum ether (1:1, *v*/*v*) during the partitioning step [53,54]. Moreover, a solid-phase extraction (SPE) approach was used in place of liquid–liquid partitioning with DCM. For instance, Luke et al. [55] added fructose, MgSO_4_, and NaCl to the original extract to separate the water from the acetone. To phase-separate mixtures of acetone/water and acetonitrile/water, Schenck et al. [54] used Na_2_SO_4_ and MgSO_4_ as drying agents. The authors discovered that acetonitrile was more successfully and efficiently separated from the water than acetone and that MgSO_4_ was more efficient in removing any remaining water from the organic layer.

Compared to other traditional techniques, the QuEChERS (quick, easy, cheap, effective, rugged, safe) method is widely used because of its many advantages, such as the ease of sample preparation, inexpensiveness, less organic solvent use, high recovery, and accuracy [56,57]. Noh et al. [58] described the QuEChERS technique as a streamlined strategy for analytical chemists to define the concentrations of multiclass and multi-residue pesticides in fruits and vegetables. With this method, MgSO_4_ was used in a new way for salting-out extraction and partitioning with acetonitrile, cleaning with dispersive solid-phase extraction (d-SPE), and detection with mass spectrometry (MS). The initial version of the QuEChERS approach demonstrated remarkable performance in detecting hundreds of pesticides in various products. Nevertheless, using the initial approach caused the poor recovery of some pH-dependent pesticides, including pymetrozine, thiabendazole, and imazalil [59]. This approach has undergone some modifications, mainly concerning pH variations and the use of a rather powerful acetate-buffered version, which became the official method of the Association of Official Analytical Chemists (AOAC) [43]. Instead, a citrate-buffered version was adopted as the European Standard (EN) procedure by the European Committee for Standardization (CEN) [60]. Due to frequent modifications of the solvents, salts, buffers, and sorbents used in the QuEChERS analytical approach, the QuEChERS approach is viewed as a sample preparation idea instead of a specific procedure [61]. Modifications are required to avoid pesticide degradation, achieve a reasonable recovery within an acceptable range, and lessen the matrix influence in complex matrices [62].

### Challenges in Sample Preparation

Several challenges can arise during the sample preparation process to determine pesticide residues in peppers. Some of these challenges include the following:

- *Sample size*: Peppers can vary significantly in size, and can be challenging to accurately sample the fruit in a way that represents the overall population.
- *Contamination*: It is crucial to avoid the contamination of the sample during the preparation process, as this can affect the accuracy of the results.
- *Pesticide distribution*: Pesticides may not be uniformly distributed on the surface of the peppers, making it difficult to sample the fruit accurately.
- *Extraction efficiency*: The efficiency of the extraction process can impact the accuracy of the results, as some pesticides may be more difficult to extract than others.
- *Matrix effects:* The presence of other compounds in the peppers (such as proteins, carbohydrates, and lipids) can interfere with the analysis process and impact the accuracy of the results.

To address these challenges, it is vital to use appropriate sampling techniques and sample preparation methods and to carefully control the conditions of the analysis process to ensure the accuracy and reliability of results.

## 4. Cleanup Procedures

Before instrumentation, samples are often purified with sorbents, such as MgSO_4_ combined with primary secondary amine (PSA), octadecylsilyl-derivatized silica (C18), and graphitized carbon black (GCB) [60,63]. The limited recovery of C18 in the analysis of nonpolar molecules and the great affinity of the GCB Table for planar analytes are two drawbacks of these often-employed sorbents. Therefore, additional efforts are needed to create novel sorbents or to optimize sorbent combinations to improve the purification effectiveness of matrices.

SPE was created to replace traditional partitioning and reduce the dangerous chlorinated solvents used in the partitioning stage [64]. The technique still needs a sizable glass column with sizable amounts of solvent for washing and elution, even though SPE was used in place of partitioning. Consequently, steps were taken to limit the consumption of solvents. The initial strategy used short florisil columns [64]. Instead of the large classical cartridges, C18 and Florisil column cartridges were also assessed in the cleanup of organo-halogen pesticides in crop matrices, and both cartridges showed acceptable recovery rates [65]. Therefore, SPE cartridges with normal or reversed-phase supports are now commercially available and provide a simple means for sample cleanup without requiring large amounts of solvent. Another extraction and cleanup method, known as matrix solid-phase dispersion (MSPD), was created to overcome the general limitations of liquid–liquid partitioning and SPE columns, including the requirement for many solvents and the emulsification of some fruits and vegetables, which blocks the flow of the analytes [66,67]. The MSPD strategy entails mixing a tiny quantity of the matrix with C18, washing it with a small amount of solvent, and eluting it to extract various chemicals (Barker, 2000a). Nonetheless, because of the minute sample size (0.5 g) used in this method, MSPD did not offer an analytical scope or a process that was adequately broad or straightforward. Anastassiades et al. [53] introduced d-SPE QuEChERS following a similar MSPD strategy [66,68,69]. The sorbent was then combined with an aliquot of the extract instead of the original sample, as in MSPD.

## 5. Instrumentation

Gas chromatography (GC), high-performance liquid chromatography (HPLC), and chromatography−mass spectrometry (GC–MS) are among the main methods that are used for pesticide residue detection and metabolite detection [11,28]. These conventional detection methods have good sensitivity, accuracy, precision, and reliability. However, they have some disadvantages, such as cumbersome sample pretreatment steps, the high cost of instruments and equipment requiring professional and technical personnel to operate them, and long detection processes. In this context, Rahman et al. [28] analyzed alachlor residues in the pepper and pepper leaves by GC and verified them through MS with pepper leaf matrix protection. They found that alachlor residues were present in both pepper and pepper leaf samples, with levels exceeding the MRL set by the Malaysian Food Regulation. The authors concluded that the consumption of peppers containing alachlor residues could pose a potential health risk to consumers. The study also highlighted the importance of the regular monitoring of pesticide residues in vegetables and fruits, as well as the need for stricter regulations on the use of pesticides in agriculture.

The main challenges in creating efficient methods for pesticide residue analysis include the low detection thresholds demanded by regulatory agencies, the variance in the polarity, volatility, and solubility of pesticides, and matrix coextraction [70]. Therefore, mass spectrometers, as a universal and more specific type of detector, began to be paired with chromatographic systems to overcome these problems [71]. In addition to improvements in the detection system, improvements in conventional sample preparation have been achieved regarding lowering the use of hazardous organic solvents, time, cost, and labor [72,73]. The QuEChERS sample preparation approach has met global acceptance and has been modified and adapted for various purposes due to its simplicity and flexibility [63,74]. However, this technique was created for gas chromatography−mass selective detection (GC–MSD) or liquid chromatography with tandem mass spectrometry (LC–MS/MS), which demand equipment that is uncommon in laboratories with only the most basic equipment [59,74].

In the quickly expanding food sector, automation in the analytical field is becoming increasingly important. Globally, strict rules and residue monitoring procedures are being created in response to consumer concerns about food safety. Due to the increased sample loads, high-throughput analytical techniques with sufficient precision and accuracy are needed.

## 6. Monitoring

The complexity of sample treatment largely depends on the matrix interferences and separation techniques, with GC and HPLC being the most common methods. Peppers typically have higher pesticide residue concentrations than other products because these compounds are constantly applied throughout the growing season. The research conducted in 2017 evaluated the levels of organochlorine pesticides in Nigerian noodles. The findings revealed that the chili peppers used in the noodles contained elevated levels of pesticide residues [41]. Several technologies have contributed to advancing the detection and monitoring of trace pesticide levels, including the GC-electron capture detector (ECD) [24,39,41,46] nitrogen phosphorus detector (NPD) [24,44], flame photometric detector (FPD) [38,43], GC−MS [30,35,36,37], and gas chromatography−tandem mass spectrometry (GC−MS/MS) due to the high selectivity, separation power, and identification capacity of MS (Table 2). The latest progress in MRMs associated with GC–MS/MS included the development of an analytical procedure that replaces traditional GC detectors. Nevertheless, due to the inadequate sensitivity for a few compounds, traditional GC detectors are still in use for SRMs [75].

To overcome inference problems, liquid chromatography with ultraviolet absorbance detection (LC-UVD) is commonly used [24,48,75]. Moreover, LC coupled with fluorescence detection (FLD) has been proposed as a promising solution to address suppression problems [42]. The need for cleanup has been decreased or eliminated. The method has been simplified, making it possible to recover all analytes in many different matrices via a single extraction and to detect them with either GC–MS/MS [75] or LC–MS/MS and ultrahigh-performance liquid chromatography−tandem mass spectrometry (UHPLC–MS/MS) [27,29,41,75].

**Table 2:** Overview of international monitoring for the analysis of pesticides in peppers.

Country	Number of Samples	Matrix	Number of Pesticides Detected	Sample Treatment	Determination Technique	Reference
China	299	Bell peppers	25 (15 OPs. 7 Pys. 3 CBs) 86. N > LOQ	Sample treatment 10 g sample + 25 mL MeCN → centrifugation (5000 rpm 5 min) → 3 g Nall vortex, upper layer sodium sulfate acetone/DCM (1:1; *v*/*v*) elution 15 mL	GC–MS	[30]
Turkey	725	Green pepper	170. 12.9% N > MRL N > LOQ	QuEChERS (Association of Official Analytical Chemists [AOAC]) (minor modification) 15 g homogenized sample + 15 mL MeOH/acetic acid (99:1, *v*/*v*) + 6 g anhydrous MgSO₄ + 1.5 g anhydrous sodium acetate, followed by vigorous shaking Centrifugation: Reconstitution in 8 mL upper MeOH + 900 mg anhydrous MgSO₄ + 150 mg PSA → dilution in 800 mL H_2_O/MeOH (95:5, *v*/*v*) + 2 mmol/L ammonium formate	LC–MS/MS	[31]
Jordan	21.11	Sweet pepper/ Pepper	113, N > MRL. 13/4	QuEChERS 10 g sample + 10 mL MeCN 1 min vigorous shaking. Centrifugation: 0.5 mL supernatant + dilution 0.5 MeCN → 10 mL formic acid (5% in MeCN) final concentration ca. 1 g/mL	LC–MS/MS	[32]
Saudi Arabia	211	Chili pepper	80. N > 100.28 N > MRL. 14	QuEChERS 15 g sample + 15 mL MeCN + 1% acetic acid + 6 g MgSO₄ + 2.5 g sodium acetate trihydrate → shaking → centrifugation (4 min), 5 mL supernatant + 750 mg MgSO₄ + 250 mg PSA + graphitized carbon → shaking (20 s) → centrifugation (4000 rpm, 5 min)	UHPLC–MS/MS GC–MS/MS	[33]
EU	91.015	Sweet peppers/ Bell peppers	821. N > MRL 2.7%	EFSA (European Food Safety Authority)		[47]
Cameroon	6 2	Chili pepper/ White pepper	198. N > MRL. 38 Chili (23.21) White (20.21)	QuEChERS 5 g powder + 5 mL Milli-Q water + 15 mL MeCN 1 min vigorous shaking. A mixture of disodium hydrogen citrate sesquihydrate (0.75 g), trisodium citrate dihydrate (1.5 g), NaCl (1.5 g), and anhydrous MgSO₄ (16 g) was agitated (3 min) on a shaker (300 rpm) → centrifugation (10,000 rpm, 5 min) 8 mL supernatant + 300 mg PSA + 900 mg MgSO₄ + 150 mg Cl8 → shaking (1 min) → centrifugation (3000 rpm, 5 min) 6 mL supernatant → LC–MS/MS 5 mL supernatant → evaporation → replaced by 5 mL hexane + 2 mL extract → GC-ECD	LC–MS/MS GC-ECD	[76]
Poland	16	Pepper	242. N > LOD. 4.	Regulation of the Ministry of Agriculture and Rural Development from 27 Nov. 2013. (DL. U.z 2013 r. NR oo, Pol, 1549) A multi-residue chromatographic method was based on residue extraction with an organic solvent and the further purification of the extract with column chromatography. The final determination of residues was performed on gas chromatographs Agilent 7890 and Agilent 6890 equipped with electron capture (ECD) and nitrogen–phosphorus detectors (NPD). Dithiocarbamate fungicides were analyzed by a spectrophotometric method based on their decomposition to CS2 in the acid environment and transfer to methyl blue, which was then analyzed with the spectrometer Unicam Helios		[34]
Serbia	3	Pepper	6. N > MRL	SPME 10 g sample + 20 mL MeOH centrifugation → supernatant volume adjusted to 100 mL with water → 10 times dilution; the extraction times varied from 30 to 60 min	GC–MS	[35]
Botswana	83	Green pepper	232. N > MRL2 N > LOD 5	The official AOAC method 15 g sample + internal standard (90 mL) → shaking (1 min) → 15 mL 1% acetic acid in MeCN + extraction salt → vigorous shaking (1 min); centrifugation (3000 rcf, 5 min, 10 °C); cleanup → 1 mL supernatant + 2 mL DSPE tube → vortex (1 min) → centrifugation (3000 rcf, 5 min). 1 mL aliquot → GC–MS/MS (packed with 400 mg styrene-divinylbenzene copolymer LiChrolut EN)	GC–MS	[36]
Saudi Arabia	160	Green pepper	23. N > LOD7 N > MRL 7	SPE 10 g sample + 20 mL acetone → homogenization (2 min); centrifugation (3000 rpm, 5 min); extraction columns conditioned with 6 mL ethyl acetate + 6 mL MeOH → 8 mL deionized H_2_O; sample loading under vacuum at 5 mL/min flow rate → vacuum for 30 min, after which the pesticides were eluted into 3 × 2 mL aliquots of ethyl acetate/acetone; the eluate was evaporated to less than 1 mL (nitrogen) (90:10, *v*/*v*); solvent exchanged to 2 mL acetone → 6 mL acetone	GC–MS	[37]
South Korea	7 8	Dried pepper leaves/ Dried red pepper	253. N > LOD 6 N > MRL 1 N > LOD 2	SPE 20 g sample + 50 mL H_2_O + 100 mL MeCN → homogenization (2 min) 20 g anhydrous NaCl + extract → vortex (3 min) → centrifugation (4000 rpm, 5 min, 4 °C); 10 mL aliquot → evaporation (35–40 °C) (nitrogen); the mixture was passed through a Florisil cartridge (conditioned with 5 mL acetone/hexane (2:8, *v*/*v*). The solvent in the mixture was evaporated → 2 mL acetone → GC, GC–MS. The mixture was passed through SPE NH₂ cartridges (1 g, 6 mL). Cartridge treated with 5 mL DCM/MeOH (8:2, *v*/*v*). The solvent in the mixture was evaporated → 2 mL MeOH → HPLC-UV	GC-NPD/ECD GC–MSD HPLC-UV	[24]
Canada	90	Green pepper	N > LOD. 316	Column chromatography partitioning 50 g sample + 250 mL MeCN/H_2_O (2:1, *v/v*) → filtration (one-half dilution with 500 mL water + 25 mL saturated NaCl) → partitioning with 2 × 50 mL DCM. The DCM was then dried by percolation through anhydrous Na₂SO₄ → isooctane was added → the sample was dried → redissolved in 5 mL isooctane, 4–5 mL concentrated sample cleaned up by Florisil column	GC-NPD/FPD	[38]
Spain	50	Pepper	31 N > LOD 35%918)	10 g sample + 50 mL ethyl acetate → homogenization (2 min) → 20 g sodium sulfate added → homogenization (1 min) → filtration (12 × 2 mL ethyl acetate) → evaporation → redissolved in 5 mL MeOH → LC–MS/MS	LC–MS/MS	[77]
China	15	Green pepper	33 N > LOD 4 N > MRL 1	SPE 50 g sample + 100 mL MeCN → oscillation (30 mL) → + 5 g NaCl → centrifugation (3000 rpm, 5 min) → MeCN layer evaporation (to 1 mL) → filtration using SPE column (Carb/NH₂) → ENVI-Carb (6 mL/500 mg) +NH₂-LC (6 mL/500 mg) + anhydrous sodium sulfate → wash with 5 mL acetone/methylbenzene (1:1, *v*/*v*) → wash four times with MeCN→ eluent evaporation → dissolve with MeCN → evaporation to 0.5 mL (nitrogen stream, 80 °C) → eluant was quantified to MeCN (1 mL)	GC-ECD/FPD	[39]
Cameroon	11	Chili pepper	20 N > LOD 58.9%	QuEChERS	GC-ECD	[46]
India	4	Chili pepper	8 N > LOD 4	Blend + extract with MeCN → re-extract with *n*-hexane layer by partitioning process → pigments removed by activated charcoal → extracts cleaned with concentrated H_2_SO_4_	GC-ECD	[78]
Slovenia	21	Pepper	214 N > LOQ 19 N > MRL 2	Cleanup by gel permeation chromatography → GC−MS sample heated two-phase system isooctane/stannous(Ⅱ) chloride in diluted HCl; carbon disulfide dissolved in the organic phase (isooctane) → GC−MS	LC–MS/MS	[45]
Venezuela	16	Red pepper	7 N > LOQ 14 N > MRL 6	200 g sample, chopped subsamples were weighed (4 g) in triplicate + 10 mL ethyl acetate/acetone (90:10, *v*/*v*) + 5 g anhydrous sodium sulfate. The solvent (organic layer) concentration was adjusted to 20 mL → GC-NPD	GC-NPD	[44]
Ghana	50	Pepper	9 N > LOQ 8 N > MRL 8	2 g sample + 5 mL MeCN → agitation (15 min) → MeCN layer decantation, MeCN layer centrifugation (3000 rpm, 2 min) → concentration to 2 mL with MeCN → GC-FPD	GC-FPD	[43]
Republic of Korea	1207	Pepper	250 N > LOQ N > MRL 12 (2003.8.5 2004.12.0 2005. 13.3%)	50 g sample + 100 mL MeCN (2 min) → homogenization → 10–15 NaCl → extract shaken vigorously → stand (30 min); 10 mL upper phase evaporation to dryness (60 °C, air stream) → next three cleanup steps repeated three times. (1) Dissolved in acetone (2 mL) → filtered through a 0.2 µm nylon Acrodisc (Whatman) → GC-NPD. MS (2) Dissolved in 20% acetone/hexane (2 mL) and loaded onto a Sep-Pak Florisil cartridge (Phenomenex) preconditioned with hexane (5 mL), → 20% acetone in hexane (5 mL). The cartridge was eluted with 20% acetone/hexane (5 mL) twice → evaporation → dissolved in 20% acetone in hexane (2 mL) → GC-ECD. (3) Dissolved in 1% MeOH/methylene chloride (2 mL) → loaded onto a Sep-Pak NH₂ cartridge (Varian) → reconditioned with 1% MeOH/methylene chloride (5 mL) twice → elution evaporation → dissolved in MeOH (5 mL) → a total of 2 mL of the 5 mL MeOH → LC-DAD, MS. Total of 3 mL of the 5 mL MeOH + 2 mL of 1% acetic acid (pH 3) → filtration (0.2 µm nylon Acrodisc) → LC-FLD	GC–MSD LC-FLD LC–MSD	[42]
Nigeria	12	Chili pepper/ Green bell pepper	13	Ultrasonic extraction 5 g sample + 2.5 g anhydrous sodium sulfate +20 mL ethyl acetate → shaking (270 rpm, 5 min) → sonication (40 °C, 20 min) → stand (5 min) → centrifugation (2500 rpm, 5 min) → supernatant concentration (1 mL, nitrogen gas) → SPE cartridge (conditioning MeOH, water, ethyl acetate) → sample extract loaded onto SPE → elution *n*-hexane/DCM (3:2, *v*/*v*) → eluate concentration (1 mL *n*-hexane, amber vial) → GC-ECD	GC-ECD	[41]
Mexico	207	Chili pepper source	1 (DPE)	European Environment Agency (EPA, 1981)	GC	[79]

## 7. Effect of Household Processing on Pesticide Residue Levels in Peppers

Several household processes can be used to reduce pesticides in fresh peppers, including washing and blanching. These processes can effectively remove or reduce the levels of pesticides on the surface of peppers, but they may not completely eliminate all residues. For instance, washing peppers thoroughly using running water can effectively remove surface contaminants, including pesticides, but it will not remove all pesticides, particularly those that have been absorbed into the pepper tissue [13]. Blanching is a process in which peppers are briefly boiled in water or steam and then cooled in ice water. This process can help loosen the pepper’s skin, making it easier to remove. It can also help to reduce the levels of pesticides on the surface of the pepper, as some pesticides may be removed during the boiling process [80]. Again, blanching may not be able to remove all pesticides, particularly those that have been absorbed into the pepper tissue. In this context, Kim et al. [80] evaluated the effects of various household processes, such as washing, blanching, frying, and drying, under different conditions (water volume, blanching time, and temperature) on residual pesticide concentrations. Both washing and blanching (in combination with high water volume and processing time) significantly reduced pesticide residue levels in the leaves and fruit of hot pepper compared with other processes [80]. It is worth considering other conditions/factors, such as selecting peppers that are grown using sustainable and organic practices to further reduce the levels of pesticides in peppers.

## 8. Dissipation Patterns and Preharvest Intervals in Peppers

Pesticides that are applied to peppers can be absorbed by the plant while also being present on the surface of the pepper fruit. The rate at which pesticides dissipate or break down can vary depending on several factors, including the type of pesticide, the application rate, the weather, and the application method. Generally, most pesticides will dissipate more quickly in warm, humid conditions and more slowly in cool, dry conditions. Pesticides applied to the surface of the pepper fruit may dissipate more quickly than those absorbed by the plant, as they are more exposed to the environment.

The dissipation behavior of pesticide residues in peppers has been investigated [81,82,83,84,85,86,87]. For instance, Liu et al. [85] reported that the t_1/2_ values of metalaxyl in peppers were 3.2–3.9 days at three experimental locations in China. At harvest, pepper samples were found to contain metalaxyl and cymoxanil levels that were well below the MRLs of the EU following the recommended dosage and an interval of 21 days after the last application.

The environmental fate of field-applied synthetic pesticides has been under investigation for several years. Endosulfan 3 EC, a mixture of α- and β-stereoisomers, was sprayed on field-grown pepper at the recommended rate of 0.44 kg of active ingredients per acre. Endosulfan sulfate is the major metabolite of endosulfan sulfite, and the β-isomers are relatively more persistent than the α-isomers. In pepper, the α-isomer, which is more toxic to mammals, dissipated faster (*t*_1/2_ = 1.22 day) than the less toxic β-isomer (t_1/2_ = 3.0 day). These results confirm the greater loss of the α-isomer than the β-isomer, which can ultimately impact endosulfan dissipation in the environment [82].

The degradation behavior of flonicamid and its metabolites, 4-(trifluoromethyl)nicotinic acid (TFNA) and *N*-(4-trifluoromethylnicotinoyl) glycine (TFNG), was evaluated in red bell peppers over 90 days under greenhouse conditions, including high temperature, low and high humidity, and in a vinyl house covered with a high-density polyethylene light shade covering film (35% and 75%). For safety reasons, the authors concluded that red bell peppers should be grown under greenhouse conditions because solar radiation increases the rate of flonicamid degradation into its metabolites [88].

It is also possible to reduce the need for pesticides by using integrated pest management techniques, such as introducing natural predators of pests or using physical barriers to prevent pests from accessing plants.

PHIs are the minimum amount of time that must pass between the application of a pesticide and the harvest of a crop. The purpose of PHIs is to allow pesticides to break down or dissipate in the environment and on the surface of the crop to levels that are considered safe for consumption. PHIs vary depending on the specific pesticide used, the type of crop, and the application method. It is important to follow the label instructions for a particular pesticide, as these will include the recommended PHI for the crop in question to ensure that the peppers are safe to consume. It is also worth noting that some pesticides may not be approved for peppers, which means there would be no recommended PHI. It is important to use pesticides only as directed and to follow all label instructions to help ensure the safety of the crop and to protect human health.

## 9. Dietary Risk Assessment

The dietary risk assessment of pesticide residues in peppers is an important task that helps determine the potential health effects of consuming peppers treated with pesticides. This assessment typically involves several steps, including:

1. Identify the pesticides that are commonly used on peppers, as well as their maximum residue levels (MRLs).
2. Collect data on the levels of pesticide residues found in peppers sold on the market.
3. Evaluate the potential health risks posed by the consumption of peppers with pesticide residues based on the levels found and the MRLs.

Once the data are collected, they can be used to estimate the average daily intake of each pesticide for different population groups. This can be performed by using data on pepper consumption patterns and the levels of pesticide residues found in peppers. Next, the potential health risks posed by the consumption of peppers with pesticide residues can be evaluated by comparing the estimated daily intake of each pesticide with the appropriate reference doses (RfDs), such as acceptable daily intakes (ADIs) or acute reference doses (ARfDs) [30,89]. These values are established by regulatory agencies, such as the US Environmental Protection Agency (EPA), as a safe level of exposure for the general population. It is worth mentioning that to have a comprehensive view of the impact of pesticide residues in peppers on human health, it is crucial to look not only at the impact of a single pesticide but also at the combined effect of different pesticides that may be present in the pepper [90,91,92]. While the impact of individual pesticides on human health has been extensively studied, the combined effect of multiple pesticides is less understood. However, there is growing evidence to suggest that exposure to multiple pesticides can have additive or synergistic effects on human health and that the cumulative effect of these residues may be greater than the effect of individual pesticides alone. Therefore, it is important to consider the potential combined impact of multiple pesticide residues when evaluating the health risks associated with consuming peppers or other fruits and vegetables. In addition, the levels of the detected pesticide in peppers can be tolerated and do not pose a serious health problem to the community [30,31]. However, it is worth noting that some people may be more sensitive to pesticides than others, such as pregnant women and children [93]. Additionally, long-term exposure to low levels of pesticides may also pose health risks [94]. It is also important to note that the risk assessment process may vary by country, as different countries have different regulations for pesticides, different exposure scenarios, and different methods for assessing risks. It is worth mentioning that regulatory agencies continuously monitor the situation and update their guidelines and regulations as necessary.

## 10. The Use of Pepper Leaf Matrix as an Analyte Protectant

The pepper leaf matrix is a complex mixture of compounds that are found in the leaves of pepper plants. It is composed of various organic compounds, such as proteins, carbohydrates, and lipids, as well as inorganic compounds, such as minerals. The specific composition of the pepper leaf matrix depends on the pepper plant variety and the growing conditions. It is possible that the pepper leaf matrix could be used as an analyte protectant during GC analysis [26,28]. Analyte protectants are substances used to stabilize or protect specific molecules or compounds during the analysis process. This can help prevent the degradation or loss of the analyte [26,28], ensuring that accurate and reliable results are obtained.

## 11. Challenges and Limitations in Managing Pesticide Residues in Peppers: Author’s Perspectives

There are several challenges and limitations when measuring and managing pesticide residues in peppers. Some of the main challenges and limitations include the following:

- *Detection limits*: Many pesticides break down or degrade over time, making it difficult to accurately measure their residues in peppers. This can be incredibly challenging when trying to detect low levels of pesticides, as the limits of detection for many analytical methods may be higher than the levels of residues present in the peppers.
- *Matrix interference*: The presence of other substances in the pepper sample, such as sugars and other organic compounds, can interfere with the accuracy of pesticide residue analysis.
- *Sample preparation*: Preparing samples for pesticide residue analysis can be time-consuming and labor-intensive. It is important to follow proper sample preparation procedures to ensure that the samples are representative and that the analysis results are accurate.
- *Regulatory limits*: Different countries and regions have different regulations and guidelines for the MRLs of pesticides in peppers and other food products. Ensuring that peppers meet these regulatory limits can be challenging, especially when dealing with multiple pesticides and different regulatory frameworks.
- *Pesticide resistance*: Some pests and diseases that affect peppers can develop resistance to certain pesticides over time. This can make it more challenging to control these pests and diseases and can lead to the need for more frequent or higher applications of pesticides.

Overall, managing pesticide residues in peppers can be a complex and challenging task. It is important to follow proper pesticide application and management practices to minimize the levels of residues in peppers and to ensure that they meet regulatory limits.

## 12. Conclusions and Future Perspectives

Depending on their use, pesticides might have positive and negative effects on peppers. It is important to use pesticides responsibly and to follow all label instructions to minimize any potential negative effects on peppers and other non-target organisms. The analytical approach to monitoring pesticide residues in peppers and their monitoring frequency is important in ensuring the safety of the food supply. Various analytical methods and sample preparation techniques are available, and regulatory agencies and food safety organizations play a crucial role in monitoring pesticide residues in peppers to ensure that they are safe for human consumption. Notably, managing pesticide residues in peppers can be a complex and challenging task. Therefore, it is essential to follow proper pesticide application and management practices to minimize the levels of residues and ensure that they meet regulatory limits. A trend toward using safer and more sustainable pest control methods in pepper production, appropriate sample cleanup methods, techniques designed to remove matrix interferences (such as pigments, lipids, and carbohydrates) and purify the target analytes effectively, and the use of accurate and reliable analytical methods should be considered. It is also important to wash and peel peppers thoroughly before consuming them to reduce the risk of exposure to pesticide residues. Overall, the outcomes of pesticide residue analysis in peppers depend on the specific method used, the type and concentration of pesticides detected, and the regulatory standards that apply.

There are several potential future developments in pesticide residue analysis in peppers that may emerge in the coming years. For instance, more sensitive and accurate methods could be developed to detect the trace levels of new pesticides. As consumer demand for organic produce grows, there may be an increased focus on alternative pest control methods that do not involve synthetic pesticides. This could lead to a decrease in the levels of pesticide residues that are found in peppers. Automating analytical techniques could also become more widespread in the future, improving the efficiency and accuracy of pesticide residue analysis in peppers. MRLs for pesticides in food, including peppers, are periodically reviewed and updated. There is an ongoing debate about what levels of pesticide residues are safe for human consumption and how MRLs should be established. Risk assessment methods are also being developed to help determine the potential health risks associated with different levels of pesticide residues in peppers.

## Figures and Tables

**Table 1:** Pesticide levels of peppers in which the maximum residue limits (MRLs) were exceeded.

Sample Matrix	Pesticide	Efficacy	Peppers MRL		References
			EU	Codex	
Bell Pepper	Bifenthrin	Insecticide, Acaricide	0.5	0.5 (P), 5 (D)	[30]
	Ethoprophos	Nematicide, Insecticide	0.05	0.2 (D)	
	Cypermethrin	Insecticide	0.5	2, (10, D)	
	Cyhalothrin	Insecticide	0.1	0.3 (F), 3 (D)	
	Carbofuran	Insecticide, Nematicide	0.002 *	—	
	Monocrotophos	Insecticide, Acaricide	0.01 *	—	
	Dimethoate	Insecticide, Acaricide	0.01 *	3 (D)	
	Methamidophos	Insecticide, Acaricide	0.01 *	—	
Green Pepper	Acetamiprid	Insecticide	0.3	0.2 (F), 2 (D)	[31]
	Boscalid	Fungicide	3	3 (F), 10 (D)	
	Azoxystrobin	Fungicide	3	3 (F), 30 (D)	
	Triadimenol	Fungicide	0.5	1 (F), (5 D)	
	Cyprodinil	Fungicide	1.5	2 (F), (9, D)	
	Metalaxyl	Fungicide	0.5 (Including Metalaxyl—M)	1 (P), (10 D)	
	Spinosad	Insecticide	2	0.3 P, (3 D)	
	Tebuconazole	Fungicide	0.6	10 (D)	
	Thiamethoxam	Insecticide	0.7	0.7 (F), (7 D)	
Pepper	Hexaconazole	Fungicide	0.01 *	—	[32]
	Propiconazole	Fungicide	0.01 *	—	
Chili pepper	Methomyl	Insecticide, Acaricide	0.04	0.7 (P), (10 D)	[33]
	Imidacloprid	Insecticide	0.9	1 (P), (10 D)	
	Metalaxyl	Fungicide	0.5 (Including Metalaxyl—M)	1 (P), (10 D)	
	Cyproconazole	Fungicide	0.05 *	—	
Greenhouse sweet pepper	Azoxystrobin	Fungicide	3	3 (F), 30 (D)	[34]
	Iprodione	Fungicide	0.01 *	—	
	Pyrimethanil	Fungicide	2	—	
Field sweet Pepper	Boscalid	Fungicide	3	3 (F), 10 (D)	[34]
Pepper	Napropamide	Herbicide	0.01 *	—	[35]
	Pendimethalin	Herbicide	0.05 *	—	
	Trifluralin	Herbicide	0.01 *	—	
	Diazinon	Insecticide, Acaricide	0.05	0.5 (D)	
	Malathion	Insecticide, Acaricide	0.02 *	0.1 (P), (1 D)	
	Pirimiphos—methyl	Insecticide, Acaricide	0.01 *	—	
Green pepper	Chlorpyrifos	Insecticide	0.01 *	20 (D)	[36]
	Chlorfenapyr	Insecticide, Acaricide	0.01 *	0.3 (P), (3 D)	
	Tebuconazole	Fungicide	0.6	10 (D)	
	Methamidophos	Insecticide, Acaricide	0.01 *	—	
	Cypermethrin	Insecticide	0.5	2, (10, D)	
Dried red pepper	Pencycuron	Fungicide	0.02 *	—	[24]
	Kresoxim—methyl	Fungicide	0.8	—	
	Diazinon	Insecticide, Acaricide	0.05	0.5 (D)	
Green pepper	Amitrole	Herbicide	0.01 *	—	[37]
	Bromoxynil	Herbicide	0.01 *	—	
	Carbaryl	Insecticide, plant growth regulator	0.01*	0.5, (2 D)	
	Carbofuran	Insecticide, nematicide	0.002 *	—	
	Chlorpyrifos	Insecticide	0.01 *	20 (D)	
	Dicofol	Acaricide	0.02 *	—	
	Malathion	Insecticide, Acaricide	0.02 *	0.1 (P), (1 D)	
	Metalaxyl	Fungicide	0.5 (Including Metalaxyl—M)	1 (P), (10 D)	
	Methoxychlor	Insecticide	0.01 *	—	
	Paraquat	Herbicide	0.02 *	0.05 (F)	
	Propoxur	Insecticide	0.05 *	—	
	Pyrethrin 1	Insecticide, Acaricide	1	0.05 (P), (0.5 D)	
	Tefluthrin	Insecticide	0.01 *	—	
	Tolclofos—methyl	Fungicide	0.01 *	—	
Green pepper	Endosulfan	Insecticide, Acaricide	0.05 *	—	[38]
	Azinphos-methyl	Insecticide	0.01 *	—	
	Carbaryl	Insecticide, plant growth regulator	0.01 *	0.5, (2 D)	
	Carbofuran	Insecticide, Nematicide	0.002 *	—	
	Chlorothalonil	Fungicide	0.01 *	7 (P), (70, D),	
Green pepper	Dichlorvos	Insecticide, Acaricide	0.01 *	—	[39]
	Omethoate	Insecticide, Acaricide	0.01 *	—	
	Ethoprophos	Nematicide, insecticide	0.05	0.2 (D)	
Red pepper	Chlorpyrifos	Insecticide	0.01 *	20 (D)	[40]
Green pepper, chili pepper	Aldrin	Insecticide	0.01 *	—	[41]
	Alpha—BHC	Insecticide	HCH	—	
	Beta—BHC	Insecticide	HCH	—	
	Chlorothalonil	Fungicide	0.01 *	7 (P), (70, D),	
	Delta—BHC	Insecticide	HCH	—	
	Dieldrin	Insecticide	0.01 *	—	
	Endosulfan	Insecticide, Acaricide	0.05 *	—	
	Heptachlor	Insecticide	0.01 *	—	
	Heptachlor—epoxide	Insecticide	0.01 *	—	
	p,p’—DDT	Insecticide	0.05 *	—	
Pepper	Chlorothalonil	Fungicide	0.01 *	7 (P), (70, D),	[42]
	Endosulfan	Insecticide, Acaricide	0.05 *	—	
	Ethoprophos	Nematicide, insecticide	0.05	0.2 (D)	
	Kresoxim—methyl	Fungicide	0.8	—	
	Pirimiphos—methyl	Insecticide, Acaricide	0.01 *	—	
	Procymidone	Fungicide	0.01 *	—	
Pepper	Methyl—chlorpyrifos	Insecticide, Acaricide	0.01 *	20 (D)	[43]
	Ethyl—chlorpyrifos	Insecticide, Acaricide	0.01 *	20 (D)	
	Dimethoate	Insecticide, Acaricide	0.01 *	3 (D)	
	Malathion	Insecticide, Acaricide	0.02 *	0.1 (P), (1 D)	
Red pepper	Methamidophos	Insecticide, Acaricide	0.01 *	—	[44]
	Diazinon	Insecticide, Acaricide	0.05	0.5 (D)	
	Dimethoate	Insecticide, Acaricide	0.01 *	3 (D)	
	Parathion—methyl	Insecticide	0.01 *	—	
	Chlorpyrifos	Insecticide	0.01 *	20 (D)	
	Malathion	Insecticide, Acaricide	0.02 *	0.1 (P), (1 D)	
Pepper	Azoxystrobin	Fungicide	3	3 (F), 30 (D)	[45]
	Cyprodinil	Fungicide	1.5	2 (F), (9, D)	
	Fludioxonil	Fungicide	1	1 (P), (4 D)	
	Lufenuron	Insecticide, Acaricide	0.8	—	
Pepper	Chlorothalonil	Fungicide	0.01 *	7 (P), (70, D),	[42]
	Endosulfan	Insecticide, Acaricide	0.05 *	—	
	Ethoprophos	Nematicide, Insecticide	0.05	0.2 (D)	
	Kresoxim—methyl	Fungicide	0.8	—	
	Pirimiphos—methyl	Insecticide, Acaricide	0.01 *	—	
	Procymidone	Fungicide	0.01 *	—	
Chili pepper	Aldrin	Insecticide	0.01 *	—	[46]
	Dieldrin	Insecticide	0.01 *	—	
	Endrin	Insecticide	0.01 *	—	
	HCB	Fungicide	0.01 *	—	
	Heptachlor	Insecticide	0.01 *	—	
	o,p’—DDT	Insecticide	0.05 *	—	
	p,p’—DDT	Insecticide	0.05 *	—	
Sweet pepper, Bell pepper	Chlorfenapyr	Insecticide, Acaricide	0.01 *	0.3 (P), (3 D)	[47]
	Triadimefon	Fungicide	0.01 *	1 (F), (5 D)	

(*) Indicates lower limit of analytical determination. (P) Group: Peppers (F) Group: FVOTC (D) Dry Pepper. FVOTC: Fruiting Vegetables, other than Cucurbits. EU: European Union.

## Data Availability

Data is contained within the article.
